# Clinical Lecture on Erysipelas

**Published:** 1860-03

**Authors:** E. Andrews

**Affiliations:** Surgeon of the Hospital, and Professor of Surgery in the Medical Department of Lind University


### CLINICAL LECTURE ON ERYSIPELAS.

Delivered at Mercy Hospital, by E. ANDREWS, M. D.,
Surgeon of the Hospital, and Professor of Surgery in the Medical Department of Lind University.

Gentlemen:

I present before you here a case of disease of a kind which will often confront you to your sorrow in your professional career, and which will occasionally bring your best planned and most brilliant surgical efforts to a disastrous termination. It is a case of traumatic erysipelas. This subject is so imperfectly understood by the profession, and so wretchedly descanted upon even by our best text books, that I have to some extent been obliged to investigate it *de novo*, and am obliged to condemn some very common precepts of the best authors. At the same time the importance of a correct knowledge of the disease is such that we may well spend our whole hour upon it.

Gentlemen, Erysipelas in some form or other, is the cause of more deaths after surgical operations and injuries, than any other single condition.

It is comparatively a rare thing in modern surgery to have a patient die from the effects of a pure and simple inflammation. Such has been the progress of our art, that we are able to cut short with promptness and great certainty almost every case of simple acute inflammation that presents itself to us. But the slightest experience shows the surgeon that when he meets a case of traumatic erysipelas, he has something more than the ordinary effect of a wound to deal with, and that there are malignant tendencies present which must be met by other measures than those of a simple antiphlogistic character.

After a full consideration of the subject, I have adopted the following definition of this scourge of surgery. *Erysipelas is an inflammation produced by the presence of a peculiar organic poison acting upon a system laboring under an aplastic diathesis.*

These three elements, the inflammation, the poison, and the aplastic diathesis, will constitute at all times an erysipelas, but if either one of them be absent, the case is not erysipelas, but something else. If the poison be absent, we have simply an inflammation with an aplastic effusion. If on the other hand the poison be present, but the aplastic diathesis be wanting, then the inflammation will be limited by plastic effusion, and will be a boil, a carbuncle, or some other circumscribed abscess.

The existence of the peculiar poison of erysipelas is proved by innumerable instances of innoculation. Before this patient came to the Hospital he poisoned no less than three members of his family. The first was his mother, who had a scratch upon her finger, which received the poison while dressing the limb. The result was a bad whitlow on the finger. The pus from this whitlow having been brought in contact with her eyes, she had an acute purulent ophthalmia. A sister of the patient having soiled her hands with the dressings, rubbed carelessly a raw pimple on the side of her face, and had as a consequence a decided erysipelas in the face. The father poisoned one of his hands in a similar manner, and had a slight erysipelas following it. A patient of a friend of mine had erysipelas in the leg which was communicated to an attendant in a similar manner, causing the death of the latter, by sloughing of the affected tissues and by pyæmia.

These instances are taken from many which have fallen under my observation, and they all go to shew the existence of this peculiar poison.

The poison existing in certain subjects in the dissecting-room is of the same character, and during several years experience as demonstrator of anatomy, I had formerly an opportunity to satisfy myself that poisoning by dissection wounds is in every instance an innoculation of erysipelas, and that it will only prove fatal when inserted into systems under an aplastic diathesis.

The erysipelatous virus is of different degrees of virulence in different cases. In some instances it only irritates the tissues enough to cause a moderate serous effusion into the areolar spaces, while in others it goes through the meshes of the supercial fascia like a solution of caustic, killing and half dissolving the tissue as it goes, and causing gangrene over the surface of half a thigh or arm at once.

That an aplastic diathesis is necessary to the existence of traumatic erysipelas is proved by a most interesting series of observations. There have occurred within the sphere of my observation many instances where different persons were inoculated at the same time, and I observe that the effect of the same virus is widely different in different persons. Thus, if the system of one is vigorously plastic, as shown by a quick healing of all cuts and scratches, an inaptitude for suppuration, and an active and healthy state of those secretions of the body which are normally acid in their reaction, such a person *cannot have erysipelas.*

If the virus is inserted into his tissues, it will be immediately circumscribed by a plastic effusion, a regular abscess will form, and the poison be ejected along with the pus. If the poison be very virulent, it will produce a slough, and a carbuncle will result.

A medical officer of the U. S. Army, informed me that he in company with another surgeon once made a post mortem examination of a child that had died from erysipelas. Both these gentlemen wounded themselves slightly in the dissection. In the case of my informant, who was in a strongly plastic diathesis, the poison was limited by plastic effusion, and the wound became the seat of a carbuncle. His companion, however, whose system was in a less favorable condition, had traumatic erysipelas in a bad form and died.

You see therefore why it is that antiphlogistics fail of their usual effects in inflammations of an erysipelatous character. While you are attempting to subdue the action by cold and purgatives and bleeding, a malignant poison is filtering through the tissues and destroying them. What we have to do in this disease is chiefly comprehended under two heads. First, to antidote the poison, and secondly, to correct the aplastic diathesis, and I am happy to inform you, gentlemen, that the means of accomplishing both these ends are now discovered.

Iodine has the power of destroying most, if not all organic poisons. Even the venom of serpents when mixed with it becomes harmless. The application of Tincture of Iodine to erysipelas is an old and favorite mode of treatment, and one worthy of all commendation. The ordinary method, however, of simply painting the affected part with the tincture two or three times a day, is by no means as decided a treatment as these cases often demand. In the present instance I shall order cotton batting *dipped* in the tincture to be wrapped around the whole arm, and confined by a piece of india rubber cloth to prevent evaporation. In this way I expect to produce a more active and complete absorption of the remedy than usual. It is probable that chlorine, bromine, and also that allotropic form of oxygen called ozone, have all antidotal powers similar to those of iodine, but as yet they are not much used for this purpose.

The second indication in this case is to effect an immediate change of the patient's diathesis from the aplastic to the plastic. Now it is becoming a favorite opinion with pathologists that the aplastic diathesis depends upon the presence of an excess of alkalies in the system, and that in particular ammonia is in excess in a variety of low adynamic forms of disease. In confirmation of this opinion is the fact that those remedies which most rapidly promote the plastic diathesis are all capable of neutralizing free alkalies. Such are the mineral acids, the soluble sulphates, iodides, chlorides, and nitrates of iron, copper, zinc, silver, and mercury. But among all these, I know of none practically so valuable for internal administration as the perchloride of iron, either in the form of a watery solution, or of the muriated tincture. With this remedy we have almost complete control over the diathesis. I have not time now to detail cases, but, gentlemen, I know what I assert when I say that with this remedy a total change in the diathesis can be made in twenty-four hours, so that at the end of that time an erysipelas shall not be able to extend to any new tissues. You must not think to accomplish this however by feebly prescribing twenty drops three times a day. I shall order this patient twenty drops every hour night and day, and such is my confidence in the result that I predict to you that to-morrow morning as we enter the ward you shall see a marked diminution of the swelling, and at the end of forty-eight hours I expect to discharge the patient convalescent. This use of the perchloride of iron is a recent advance in surgery, and one of the most valuable improvements ever made. It is only in the early stages of the disease that you can predict its success with the confidence which I have done in this case. After suppuration has already occurred, an organic mischief has been done which time alone can repair, although the remedy is still useful to prevent further destruction.

That domestic remedy, the cranberry poultice, is probably useful, correcting by its acid the excess of alkali in the diseased part, and so promoting plasticity. I caution you, however, against expecting any plastic result from the *internal* use of vegetable acids. In the stomach they are all digested and destroyed, leaving no acid product.

Finally, gentlemen, in the low forms of this disease, never give ammonia as a stimulant. The system is already dissolving and breaking down into alkaline pus and serum, and every drop of ammonia adds to the fatal excess of alkali which produces the morbid result.

